# Melanin pigments in the melanocytic nevus regress spontaneously after inactivation by high hydrostatic pressure

**DOI:** 10.1371/journal.pone.0186958

**Published:** 2017-11-01

**Authors:** Michiharu Sakamoto, Naoki Morimoto, Chizuru Jinno, Atsushi Mahara, Shuichi Ogino, Shigehiko Suzuki, Kenji Kusumoto, Tetsuji Yamaoka

**Affiliations:** 1 Department of Plastic and Reconstructive Surgery, Graduate School of Medicine, Kyoto University, Kyoto, Japan; 2 Department of Plastic and Reconstructive Surgery, Kansai Medical University, Hirakata, Osaka, Japan; 3 Department of Biomedical Engineering, National Cerebral and Cardiovascular Center Research Institute, Suita, Osaka, Japan; Kyoto Daigaku, JAPAN

## Abstract

We report a novel treatment for giant congenital melanocytic nevi (GCMN) that involves the reuse of resected nevus tissue after high hydrostatic pressurization (HHP). However, the remaining melanin pigments in the inactivated nevus tissue pose a problem; therefore, we performed a long-term observation of the color change of inactivated nevus tissue after HHP. Pressurized nevus specimens (200 MPa group, n = 9) and non-pressurized nevus tissues (control group, n = 9) were subcutaneously implanted into nude mice (BALB/c-nu) and then harvested 3, 6, and 12 months later. Color changes of the nevus specimens were evaluated. In the 200 MPa group, the specimen color gradually regressed and turned white, and brightness values were significantly higher in the 200 MPa group than in the control group after 6 months. This indicated that melanin pigments in the pressurized nevus tissue had spontaneously degraded and regressed. Therefore, it is not necessary to remove melanin pigments in HHP-treated nevus tissue.

## Introduction

Congenital melanocytic nevi (CMN) are large brown-to-black skin lesions that appear at birth. Giant congenital melanocytic nevi (GCMN) with a diameter >20 cm [1–5] occur in approximately 1 out of every 20,000 newborns [1,3] and are associated with the risk of malignant transformation to malignant melanoma. The incidence of malignant melanoma from GCMN has been reported to be 0.7–8.2% [1,4]. Nevus cells are present throughout the layer of the dermis; therefore, the entire nevus tissue should be removed to prevent the emergence of melanoma [1–5]. In Japan, a cultured epidermal autograft (CEA) using Green’s method was approved in 2016, and it is currently covered by public healthcare insurance for use in the treatment of GCMN; however, an approach for the reconstruction of the dermal layer has not been established. Furthermore, the take rate of the Japanese CEA product (JACE^®^; Japan Tissue Engineering Co., Ltd., Gamagori, Japan) applied to a dermal layer reconstructed with an allograft or bilayered artificial dermis is unsatisfactory [6].

To overcome these issues, we developed a novel treatment for GCMN involving the reuse of the autologous nevus without discarding the nevus tissue [7–13]. We inactivated the removed nevus tissue using high hydrostatic pressurization (HHP) at 200 MPa for 10 minutes and autografted the inactivated nevus to the original site for dermal reconstruction. Then, we applied CEA to the inactivated nevus for epidermal reconstruction 2 or 3 weeks after grafting. Previous studies have shown that all kinds of cells in the human skin, porcine skin, and nevus tissue were completely inactivated after HHP at >200 MPa for 10 minutes [7–11]. Furthermore, the cultured epidermis survived on the inactivated skin and nevus with HHP [7,8].

An important issue with our novel treatment involves the remaining melanin pigments in the inactivated nevus tissue. Melanin pigments in nevus tissue are produced by nevus cells; therefore, we anticipated that melanin pigments remaining in the inactivated nevus tissue would regress with time spontaneously in vivo. In this study, we inactivated nevus tissue at 200 MPa and implanted it subcutaneously in nude mice. We collected specimens at 3, 6, and 12 months after implantation and observed the color changes and histology with time.

## Materials and methods

### Ethics statement

Our protocol was approved by the Ethics Committee of Kyoto University Graduate School and Faculty of Medicine (permit no. E1050). Regarding animal research, our experimental protocol was approved by the Animal Research Committee of Kyoto University Graduate School of Medicine (permit no. Med Kyo 15148). The number of animals used in this study was kept to a minimum, and all possible efforts were made to reduce their suffering in compliance with the protocols established by the Animal Research Committee.

### Preparation of nevus tissue

Nevus tissue specimens were obtained from a female patient who underwent resection surgery to remove nevi at Kyoto University Hospital. She provided written informed consent before specimens were obtained. Nevus tissues with identical texture and color were obtained from her abdominal region and thigh and were used for this study. The specimens were subjected to the HHP procedure and were used during an animal implantation study at Kyoto University. After subcutaneous adipose tissues were removed with scissors, the resected nevus tissues were immersed in normal saline solution (NSS; Otsuka Pharmaceutical Co., Ltd, Tokyo, Japan) to prevent drying.

### Inactivation of nevus specimens using an HHP device

A portable HHP device that was jointly developed by Kitaoka Iron Works Co., Ltd. (Osaka, Japan) and our team [11] was used in this study. This system consists of a main pressure unit with a hydraulic hand pump, an electric pressure generation unit, and a pressure control unit. During the HHP process, a pressure-tight cell was pressed with a piston via the electric compressor or hand pump. A pressure up to 280 MPa can be applied with this device.

Eighteen square (1 × 1 cm) nevus specimens were prepared from the resected nevus tissues. They were divided into the following two groups: control group (n = 9) and 200 MPa group (n = 9). The nevus specimens in the 200 MPa group were packed in a plastic bag filled with NSS and pressurized at 200 MPa for 10 minutes. After pressurization, the epidermis layers of the pressurized nevus tissues were detached from the dermis layers, and they were completely removed using forceps. The nevus specimens in the control group were packed in an equivalent plastic bag filled with NSS and preserved at room temperature without pressurization. The nevus tissues of both groups were immersed in NSS again and preserved at room temperature until grafting.

Digital photographs of the nevus tissues of both groups were evaluated using a standard color chart (CASMATCH^®^; BEAR Medic Corporation, Tokyo, Japan). The standard color chart was used to adjust and calibrate tones of each nevus color for analysis.

### Implantation of nevus tissue into nude mice

A total of nine nude mice (male; 5 weeks old; BALB/c-nu; Charles River Laboratories International, Inc., Yokohama, Japan) were prepared and maintained under specific pathogen-free conditions. A maximum of six mice were housed in a cage, and they had *ad libitum* access to sterile regular chow and water. Under general anesthesia (inhalation of 1–1.5% isoflurane; Pfizer Japan Inc., Tokyo, Japan), the dorsum was incised. Two nevus specimens were implanted into the subcutis of each mouse. A nevus specimen from the 200 MPa group was implanted on the right side, and a nevus specimen from the control group was implanted on the left side. The dorsum was sutured with 5–0 nylon (Diadem; Medical U&A, Inc., Osaka, Japan). Three mice were killed with exposure to carbon dioxide gas at 3, 6, and 12 months after implantation, and the implanted nevus specimens were harvested. Photographs of the nevus specimens were obtained, and a standard color chart was used. The specimens were then fixed with 10% neutral buffered formalin solution, embedded in paraffin blocks, and cut into 5-μm-thick sections. The sections were subjected to hematoxylin and eosin staining and immunohistochemical staining.

### Evaluation of the nevus color change

The HSB (hue, saturation, and brightness) color model is a representative color system used to describe color space. It consists of the following three components: hue, the color type (red, blue, or yellow); saturation, the intensity of the color; and brightness, the brilliance of the color. Brightness ranges from 0 to 100 (0 is black and 100 is the brightest version of the color in the given hue and saturation). We used the parameter of brightness to evaluate color change in the nevus specimens.

First, we adjusted the tones of all digital images. Then, we evaluated the brightness of the nevus specimens using Adobe Photoshop Elements (version 13; Adobe Systems Inc., CA). The HSB color mode was selected, and brightness was evaluated using a color picker at nine arbitrary points of each specimen. The mean brightness value at the nine points was used for analysis.

### Immunohistological evaluation of residual human cells and capillaries in grafts

Immunohistochemical staining of human vimentin was performed to detect all residual human cells, including nevus cells and fibroblasts, in grafts. Immunohistochemical staining of CD31 was performed to detect capillaries in grafts. For anti-human vimentin staining, a labeled streptavidin biotinylated antibody method was used as follows. After deparaffinization, rehydration, and antigen retrieval using the heat-induced target retrieval method, endogenous biotin activity was blocked using a biotin-blocking system (Dako X0590; Agilent Technologies, Santa Clara, CA) and non-specific staining was inhibited using a protein-blocking agent (Protein Block Serum-Free X0909, Agilent Technologies, Santa Clara, CA). Subsequently, anti-vimentin antibody (Dako M0725; Agilent Technologies, Santa Clara, CA) at a dilution of 1:100 was applied for 30 minutes at room temperature. The slides were then incubated with biotinylated goat anti-mouse immunoglobulin (Dako E0433; Agilent Technologies, Santa Clara, CA) as a secondary antibody diluted to 1:300 in PBS for 30 minutes. After reaction with enzyme-labeled streptavidin (416012, Nichirei Bioscience Inc., Tokyo, Japan), the coloring reaction was performed with permanent red (Dako K0640; Agilent Technologies, Santa Clara, CA) and nuclei were counterstained with hematoxylin.

Anti-CD31 staining after deparaffinization, rehydration, and the antigen retrieval process, was performed as described. Endogenous peroxidase activity was blocked using 3% H_2_O_2_ for 10 minutes according to the manufacturer’s instruction. Rabbit polyclonal anti-CD31 antibody (dilution 1:100; E11114; Spring Bioscience, Inc., Pleasanton, CA) was used as the primary antibody, and HRP-labeled polymer anti-rabbit antibody (EnVision System, Dako Japan Co., Ltd., Tokyo, Japan) was used as the secondary antibody. The coloring reaction was performed with DAB (3,3′-diaminobenzidine tetrahydrochloride; Nichirei Bioscience Inc., Tokyo, Japan). Microphotographs were obtained using an optical microscope (BZ-9000; KEYENCE Japan, Osaka, Japan).

### Statistical analysis

Statistical significance was assessed using Welch’s *t*-test. All data are expressed as mean ± standard deviation. The Microsoft Excel software program (Microsoft Corp., Redmond, WA) with the Statcel software add-on (OMS Publishing, Inc., Tokyo, Japan) was used for all statistical analyses. A p-value <0.05 was considered statistically significant.

## Results

### Color change in nevus specimens after grafting

**Fig 1:**
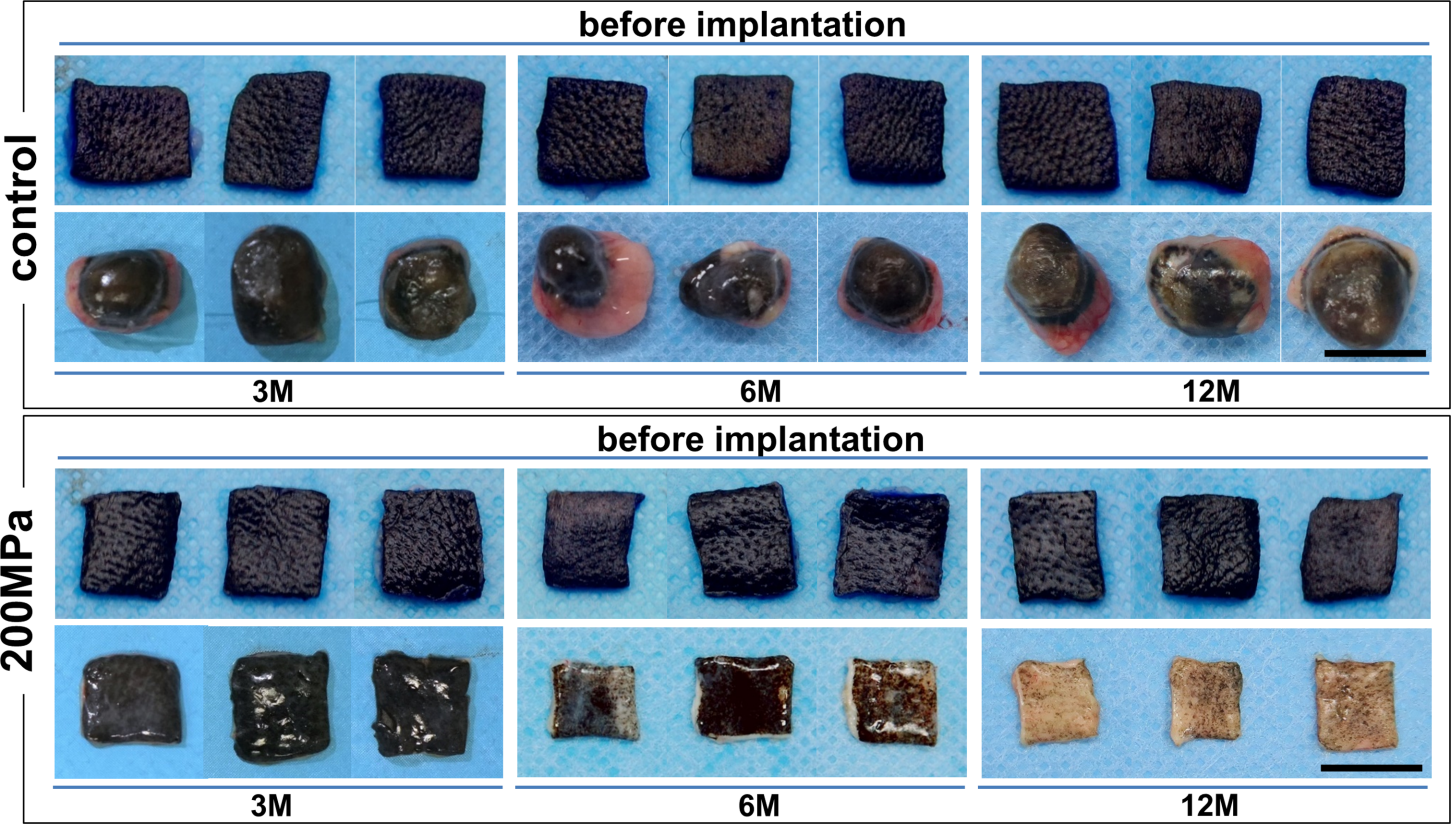
Photographs of the nevus specimens before implantation and 3, 6, and 12 months later in the control and 200 MPa groups. The nevus specimens in the control group 3 or more months after implantation show cysts and conversion to spherical forms. The color of nevus tissues in the 200 MPa group shows a gradual change to white.

**Fig 2:**
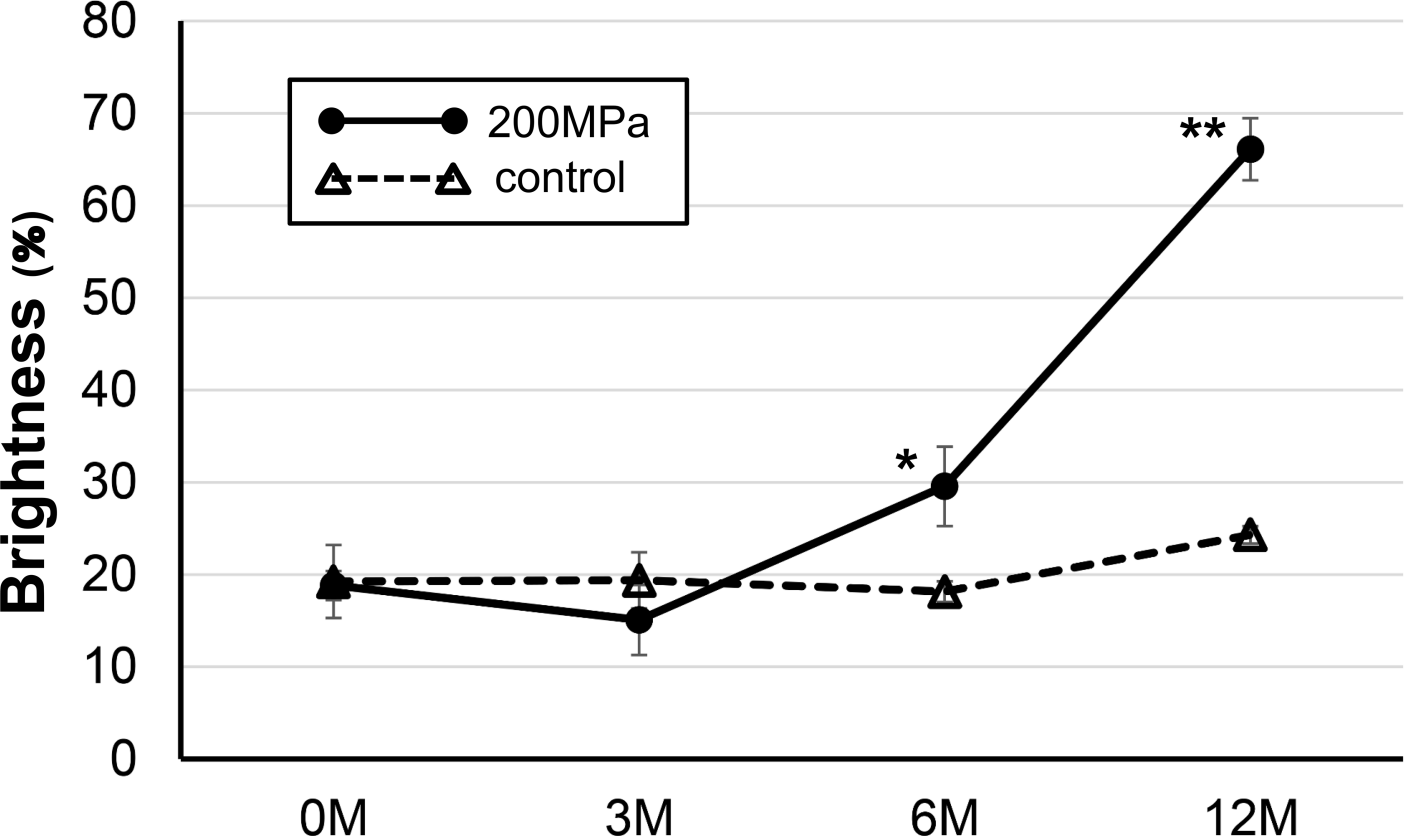
The mean brightness values of nevus specimens in the control and 200 MPa groups. The brightness values at 6 and 12 months are significantly higher in the 200 MPa group than in the control group (*p<0.05, **p<0.01).

Gross appearances of pressurized nevus specimens before and after implantation with time are shown in Fig 1. Nevus specimens in the control group after 3 months had a spherical shape and showed epidermal cysts. The color of the superficial aspect remained black. In contrast, specimens from the 200 MPa group remained flat and square until 12 months. The color of those from the 200 MPa group gradually regressed and turned white with time. The time courses of the brightness values for the control and 200 MPa groups are shown in Fig 2. The brightness values of the 200 MPa group increased after 6 months and were significantly higher than those of the control group.

### Histological evaluation of nevus specimens

**Fig 3:**
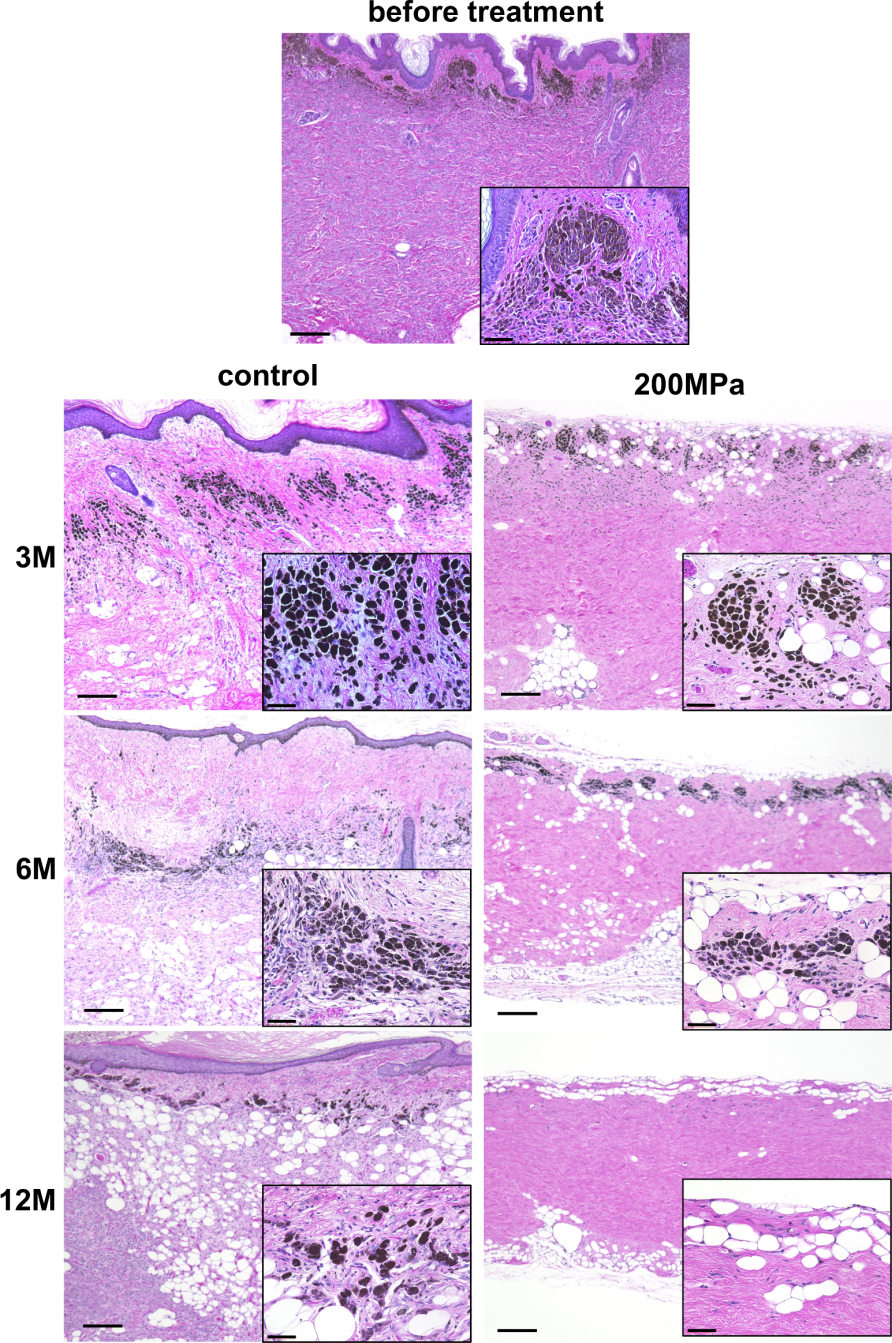
Microphotographs of sections of nevus tissue stained with hematoxylin and eosin before treatment and 3, 6, and 12 months after implantation in the control and 200 MPa groups. Scale: 200 μm (high-magnification images in the lower right corner; scale: 50 μm). In the control group, melanin pigments are seen at 12 months. In contrast, in the 200 MPa group, melanin granules at 6 months appear pale and small. Twelve months later, no large melanin granule is observed, except for few subtle granules.

Microphotographs of hematoxylin and eosin-stained sections of nevus tissue before pressurization and after implantation are shown in Fig 3. Before pressurization, melanin pigments were observed in the superficial part of the dermis and nests of nevus cells were observed in the entire layer of the dermis, representing the histological characteristics of the compound nevus. In the control group, the epidermis and melanin pigments remained until 12 months. In contrast, in the 200 MPa group, the epidermis was removed just after pressurization and no epidermis was observed at any point after implantation. Melanin granules existed at 3 months as well as before pressurization; however, they decreased in size and mostly disappeared by 12 months. No apparent inflammatory response, such as infiltration of macrophages, was observed during the observation period.

### Immunohistochemical evaluation of residual human cells and capillaries in grafts

**Fig 4:**
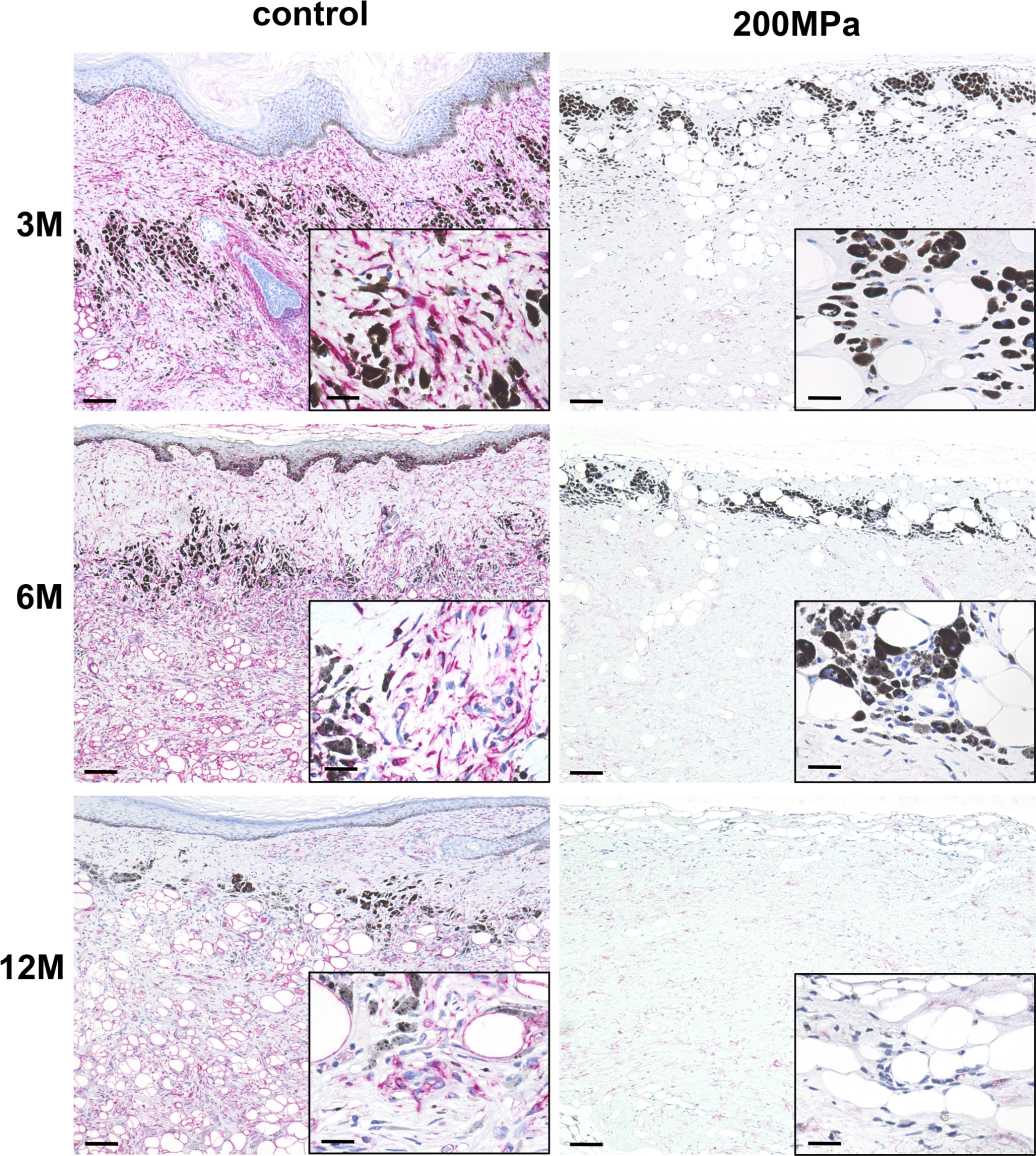
Micrographs of immunohistochemical staining of anti-human vimentin sections of nevus specimens 3, 6, and 12 months after implantation in the control and 200 MPa groups. Scale: 200 μm (high-magnification images in the lower right corner; scale: 50 μm). Anti-human vimentin sections from the control group show abundant remaining human cells stained with fast red. No human cells are noted in the 200 MPa group.

Immunohistochemical staining for human vimentin showed abundant remaining human cells in the dermal part in the control group, but no human cells were noted in the 200 MPa group at 3, 6, and 12 months (Fig 4). This indicated that human cells in grafts of the control group survived up to 12 months after grafting. On the contrary, human cells were inactivated completely after pressurization and never regrew in the 200 MPa group.

**Fig 5:**
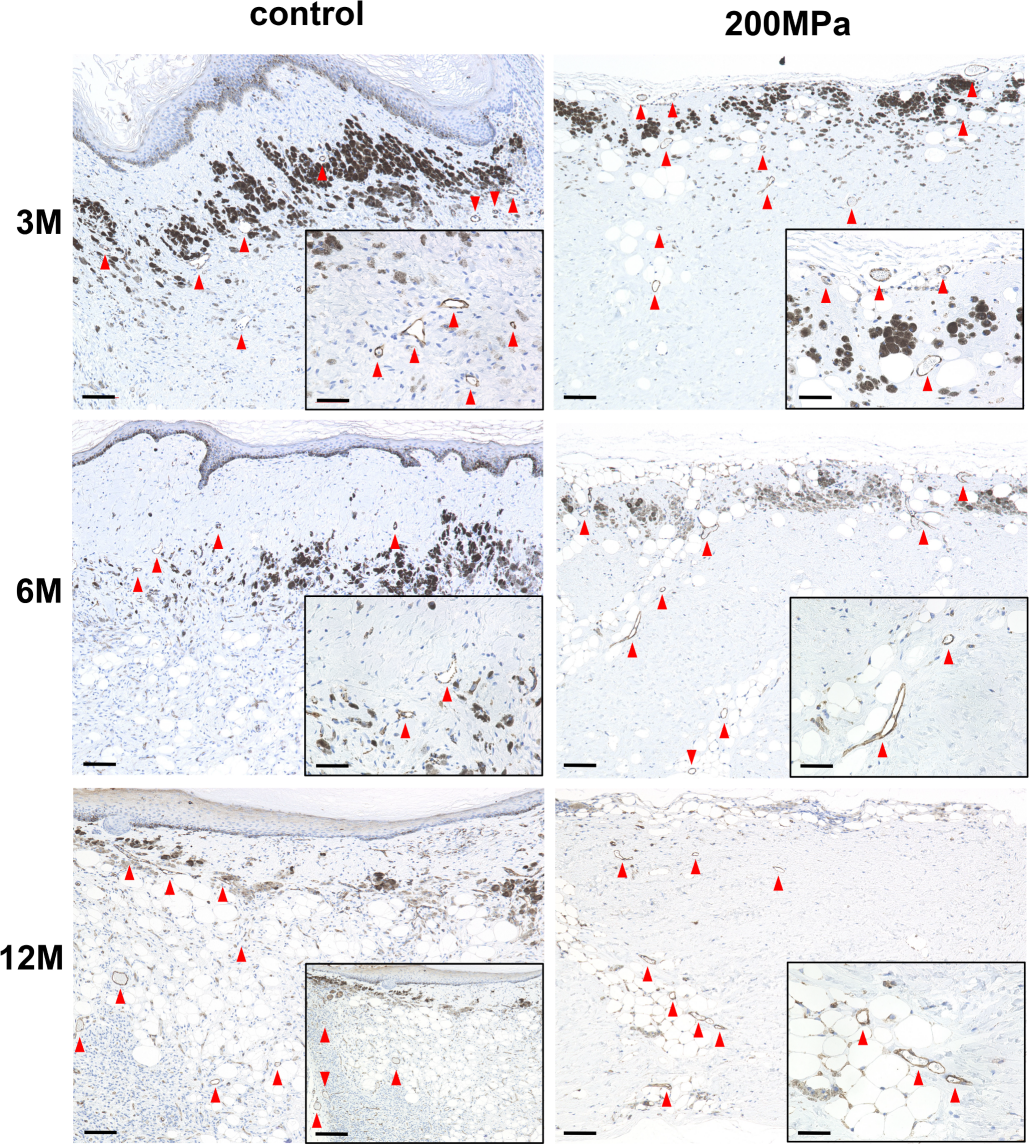
Microphotographs of immunohistochemical staining of anti-CD31 sections of nevus tissues 3, 6, and 12 months after implantation in the control and 200 MPa groups. Scale: 200 μm (high-magnification images in the lower right corner; scale: 50 μm). Regenerated capillaries (red arrowheads) are observed in the dermal parts of nevus specimens in both groups 3 or more months after implantation.

Immunohistochemical staining for CD31 showed regenerated capillaries in the dermal parts of nevus specimens in both groups after 3 months (Fig 5). This indicated that the implanted nevus tissues were revascularized and survived after grafting.

## Discussion

As described previously, CEA was approved in 2016 and is covered by public healthcare insurance for use in the treatment of GCMN in Japan. CEA has been used mainly in the treatment of severe burns worldwide since the 1990s. However, an approach for the reconstruction of the dermal layer has not been established. The Japanese CEA product (JACE^®^) was approved for the treatment of severe burns in 2007 in Japan, as the first medical device using autologous cells. JACE^®^ has been used for more than 500 burn victims, and no severe adverse events have been reported [6,14]. However, its take rate is not satisfactory, mainly because regeneration of the dermal component of the recipient site for CEA application has not been established [6,14].

An allogeneic skin graft is the most standard procedure for dermal regeneration; however, the supply of allogeneic skin has been critically limited in Japan [6]. A previous study reported that allogeneic skin accelerates the formation of the dermal component and that it does not survive at the recipient site owing to rejection [14]. The human acellular dermal matrix (HADM) is an allogeneic dermis in which all cellular elements that can lead to tissue rejection are removed [15,16]. HADM is expected to be used for the dermal reconstruction of the recipient site of CEA; however, only one case report on successful combination therapy using CEA and HADM has been published to date [17]. In our previous study using a porcine model, we showed that acellular allogeneic dermis biodegraded severely in vivo when compared with pressurized autologous dermis [12]. Therefore, we believe that the inactivated nevus is the patient’s autologous tissue and that it has the potential to survive and serve as autologous dermis without inflammation or rejection.

Concerns regarding the use of the nevus inactivated by HHP for autologous dermal reconstruction in combination with CEA involve the remaining cellular debris and melanin pigments. With regard to the remaining cellular debris, we have already reported that cellular debris biodegraded within several weeks after grafting and that CEA survived successfully on the inactivated human skin and human nevus tissue without serious inflammation [7,8,12]. On the other hand, with regard to the remaining melanin pigments, the color of the nevus tissue did not change after HHP treatment; thus, no aesthetic improvement may be provided.

Melanins represent a group of complex pigments with relatively diverse and undefined structures [18,19]. Liu et al. reported that they could remove all cellular components and melanin pigments from human nevus tissue using dispase, Triton X-100, and H_2_O_2_ [20]; however, it is usually difficult to remove melanin pigments completely without damaging the extracellular matrix [21,22]. In addition, the surfactant damages the dermal matrix and inhibits the attachment of keratinocytes [21]. Therefore, this chemical treatment is not appropriate for our HHP combination treatment.

Meanwhile, melanin pigments are known to be degraded by the action of macrophages both in vitro [23] and in vivo [24]; therefore, they are expected to regress and disappear when the biosynthesis by nevus cells stops. In fact, Mongolian spots (dermal melanocytoses with some dermal dendritic melanocytes) usually disappear with age [25,26]. Furthermore, some cases of spontaneous lightening and regression of CMN have been reported [27–29]. Based on these facts, we anticipated that melanin pigments remaining in the inactivated nevus tissue would regress with time spontaneously after implantation. Thus, our data clearly proved how quickly intrinsic melanin in the nevus inactivated by HHP was degraded in vivo macroscopically and histologically.

The implanted nevus specimens in the control group generated epidermal cysts. When viable epidermis is present in the subcutaneous space, the proliferating keratinocytes in the edge of the epidermis grow upward to generate a cyst because of the lack of space. This is a common phenomenon experienced in clinical situations. On the other hand, the implanted nevus specimens in the 200 MPa group remained flat and square, and no cysts were noted, indicating the absence of viable keratinocytes in the HHP-treated tissue, as was histologically confirmed in the HE-stained section.

Cell infiltration and revascularization are important when the inactivated nevus tissue is grafted to reconstruct the dermal component. With regard to the infiltration of recipient cells to the grafted inactivated dermis, we have already shown that recipient cells successfully infiltrated the inactivated normal skin that was implanted at the subcutaneous region within one week after grafting in an autologous porcine model [10,12,13]. In the current study, many cells were observed in the HE-stained sections in both the control and 200 MPa groups. Immunohistochemical analysis with anti-human vimentin staining was performed to determine whether these cells are human donor cells or rodent recipient cells. The anti-human vimentin antibody used in this study does not show cross-reactivity against mouse cells; thus, only human cells are stained with fast red. No human cells stained with anti-human vimentin antibody were observed at 12 months after implantation in the 200 MPa group, indicating that HHP treatment with 200 MPa for 10 minutes completely inactivated all cells in the nevus tissue. The cells that were not stained with anti-human vimentin antibody were mouse recipient cells, such as fibroblasts and endothelial cells. Thus, the inactivated nevus tissue was infiltrated with recipient cells and was revascularized as observed in the immunochemical analysis with anti-CD31 antibody. These findings suggest that the inactivated nevus tissue can regenerate viable dermal components with blood supply and infiltrated living cells after its implantation.

## Conclusion

There was no recurrence of nevus cells during the observation period, and it was determined that melanin pigments of nevus tissue will regress spontaneously in vivo after inactivation by HHP. This suggests that we can reimplant the removed and inactivated nevus without removing melanin pigments in combination with consecutive grafting of CEA. Then, the remaining melanin will regress spontaneously after grafting. Our novel treatment for GCMN using HHP is a simple and safe procedure that requires only 10 minutes and does not use chemicals. Therefore, it could be a promising method for GCMN.
